# An overview of the visual dysfunctions in Parkinson’s disease – Update from a prospective study in Transylvania

**DOI:** 10.22336/rjo.2022.28

**Published:** 2022

**Authors:** Vlad-Ioan Suciu, Corina-Iuliana Suciu, Simona Delia Nicoară, Lăcrămioara Perju-Dumbravă

**Affiliations:** * Department of Neuroscience, “Iuliu Haţieganu” University of Medicine and Pharmacy, Cluj-Napoca, Romania; ** Department of Ophthalmology, “Iuliu Haţieganu” University of Medicine and Pharmacy, Cluj-Napoca, Romania

**Keywords:** Parkinson’s disease, visual dysfunctions, multidisciplinary approach

## Abstract

Being a highly prevalent neurodegenerative disorder worldwide, Parkinson’s disease (PD) shows its complexity not only in the variability of its pathology, but also in the complex constellation of its clinical picture. PD does not affect only the nervous system; instead, it is today recognized as having both motor and non-motor features.

The purpose of this article is to report up-to-date information of an ongoing study, which correlates the motor features of Parkinson’s disease (PD) with the visual disturbances (non-motor features) related to this disease.

**Abbreviations:** PD = Parkinson’s disease, HY = Hoehn-Yahr scale, MMSE = Mini-Mental state exam, UPDRS = Unified Parkinson’s disease rating scale, RE = right eye, LE = left eye

## Introduction

Parkinson’s disease (PD) is a complex condition that affects not only the dopaminergic system, but has a wide range of systemic implications. The intricate pathological processes in PD begin decades before the motor signs are evident. The clinician must actively screen for the non-motor signs in order to timely diagnose PD and prevent disability. A proper screening for non-motor symptoms is done by a multidisciplinary patient approach and team-work [**1**-**4**].

This paper presents new data from the ongoing study that investigates the visual disturbances in PD, taking place in Transylvania region, Romania.

## Materials and methods

This is an updated report on the ongoing study that focuses on the visual disturbances in PD. This prospective, observational study recruited PD patients from Cluj County, Transylvania region, Romania, with non-advanced stages of disease. Severe ophthalmologic diseases were screened and excluded prior to recruitment. This sequel report brings new insights on the correlations between motor features and visual disturbances in a PD cohort.

## Results

All twenty-five PD subjects enrolled met the criteria of being classified in non-advanced PD stages and had no severe ocular diseases. All subjects were investigated by the same medical team: ophthalmologist and neurologist, in order to control examination subjectivity.

**Fig. 1:**
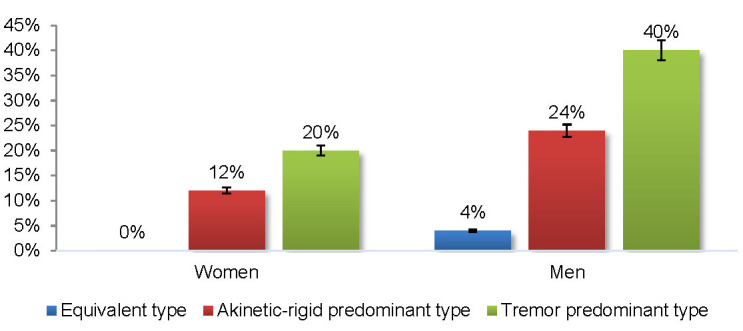
The distribution by PD subtype and gender

The distribution of patients according to PD subtypes and gender revealed a similar proportion of the total number of men and women being classified in the akinetic-rigid (women 37.5%; men 35.29%) or tremor (women 62.5%; men 58.82%) predominant type. When analyzed apart, the women population had 12% of the individuals classified in the akinetic-rigid type and 20% in the tremor type, while the male population had 24% of the individuals classified in the akinetic-rigid type and 40% in the tremor type (**Fig. 1**).

**Fig. 2:**
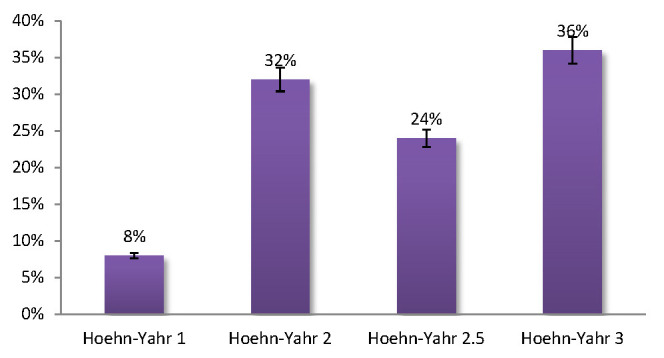
The cohort distribution according to the HY scale

When analyzing the cohort distribution according to the HY scale, the fewest subjects were classified in the first HY grade. The HY grades 2 (32%), 2.5 (24%) and 3 (36%) had similar distributions (**Fig. 2**).

**Fig. 3:**
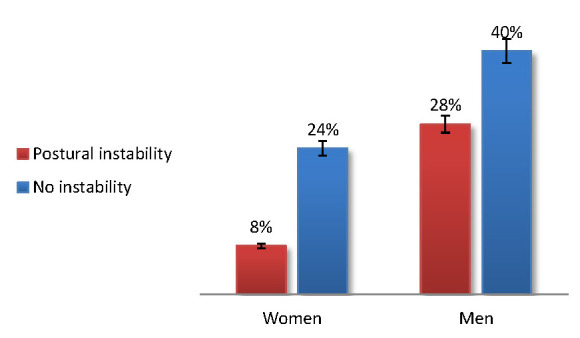
The postural instability according to the gender

36% presented some kind of postural instability, while 64% had no instability. When comparing both genders in relation to the postural instability, fewer women (-20%) presented postural instability than men. The female gender showed fewer individuals with instability (8%) than those without instability (24%) (**Fig. 3**).

**Fig. 4:**
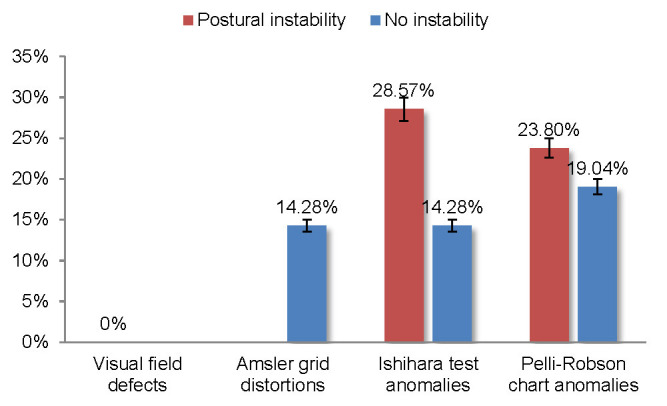
The relationship between postural stability and visual disturbances

The visual disturbances showed different incidence rates among subjects with and without postural instability. A significantly higher rate of visual disturbances was seen in the Ishihara test (+14.29% with *p* = 0.027) among the subjects with postural instability (28.57%) when compared with those with no instability (14.28%). Regarding the contrast sensitivity tests, no significant differences between the two groups (+4.76% with *p* = 0.068) were observed. No subject in this study had visual field defects (**Fig. 4**).

**Fig. 5:**
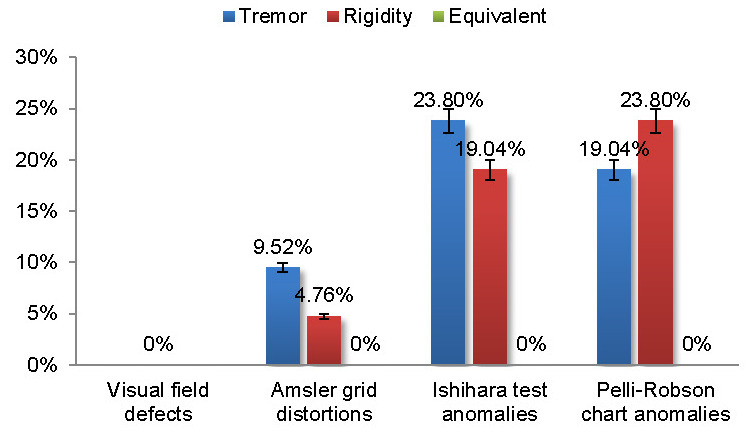
The relationship between the PD subtypes and the visual chart evaluations

The Amsler grid distortions and Ishihara test anomalies were slightly higher in the tremor predominant type when compared to the akinetic-rigid type, while the Pelli-Robson chart anomalies were higher in the akinetic-rigid type (**Fig. 5**).

The akinetic-rigid (*r* = 0,741) subtype correlated with the contrast disturbances, while the tremor predominant type had an inverse correlation (*r* = -0,769) with the contrast disturbances.

The multiple regression analysis between the Amsler grid evaluation and the HY stage (*p* = 0,048) and UPDRS-III (*p* = 0,011) score showed statistically significant results (F value 1.3868). 

The mean PD disease duration (in years) was similar between 40-59 (8.64 y), 60-69 (8.96 y) and 70-79 (9.28 y) years’ age groups. The mean disease duration correlated well with the color vision anomalies (*r* = 0.99) and with anomalies in the posterior pole of both eyes (*r* = 0.962). Also, the posterior pole anomalies of both eyes correlated well with the color vision anomalies (*r* = 0.949).

Another observation regarding the lateralization of the motor symptoms revealed that right-predominant parkinsonism was correlated with the color vision anomalies (*r* = 0,950), and with anomalies of both eyes in the ophthalmoscopic evaluation (*r* = 0,862). On the other hand, it had a strong inverse correlation with the contrast disturbances (*r* = -0,773). Left-predominant parkinsonism was correlated with contrast disturbances (*r* = 0,773) and had inverse correlation with the color vision anomalies (*r* = -0,950) and the anomalies of both eyes in the ophthalmoscopic evaluation (*r* = -0,862).

The subjects classified in the HY motor scale 3 correlated well with the color vision anomalies (*r* = 0,971) and the anomalies of both eyes in the ophthalmoscopic evaluations (*r* = 0,980), while the HY scale 1 (*r* = 0,690) and 2 (*r* = 0,827) correlated with the contrast disturbances.

Each subject was sorted by convention into two groups: UPDRS-III scores < 30 points and UPDRS-III scores ≥ 30 points. The patients with UPDRS-III scores < 30 p. correlated well with the contrast disturbances (*r* = 0,814), while those with scores ≥ 30 p. correlated well with the color vision anomalies (*r* = 0,972) and the anomalies of both eyes in the ophthalmoscopic evaluations (*r* = 0,954).

Regarding the MMSE scale, most of the patients were observed in the 30-29 p MMSE class (48%), while the fewest were classified in the 26-25 (4%) and 24-23 (4%) p MMSE class (**Table 1**).

**Table 1:** The MMSE classes and visual dysfunction tests

MMSE class	Amsler grid distortions	Color vision anomalies	Contrast sensitivity anomalies	ttest *p* Value Amsler vs. Color anomalies	ttest *p* Value Color vs. Contrast anomalies	ttest *p* Value Amsler vs. Contrast anomalies
30-29 p	12%	20%	16%	0.05*	0.50	0.09
28-27 p	0%	12%	16%			
26-25 p	0%	4%	0%			
24-23 p	0%	0%	4%			

**Fig. 6:**
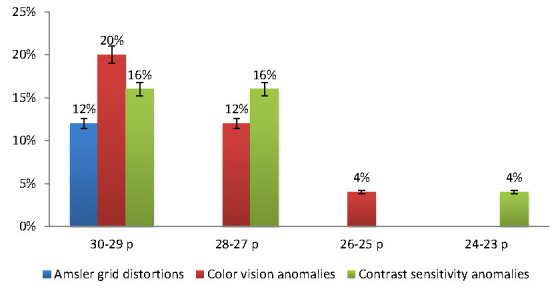
The visual dysfunctions in relation to the MMSE scale groups

Visual dysfunctions were observed in all MMSE classes. In the 30-29 p MMSE class, the highest incidence of visual dysfunctions was observed among all domains (12% Amsler grid distortions, 20% Color vision anomalies and 16% Contrast sensitivity anomalies). In the 24-23 p MMSE class, only contrast sensitivity anomalies were noted (**Fig. 6**).

The ttest for independent samples showed significant results regarding the visual dysfunctions, between the 30-29 p MMSE group and two groups: the 26-25 p MMSE group (*p* = 0.004), and the 24-23 p MMSE group (*p* = 0.002). 

**Fig. 7:**
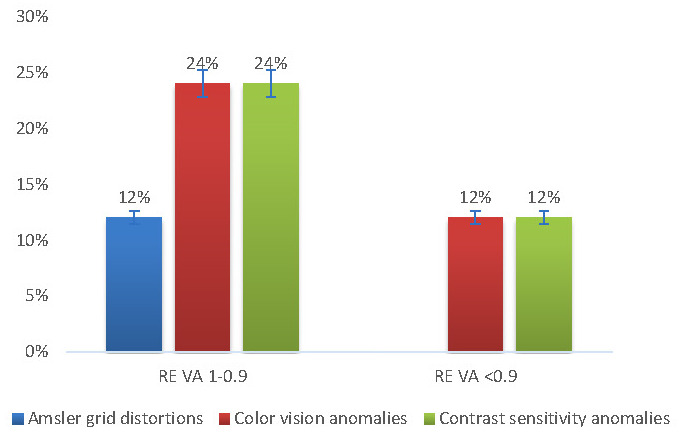
Right eye vision anomalies in relation to the visual acuity

**Fig. 8:**
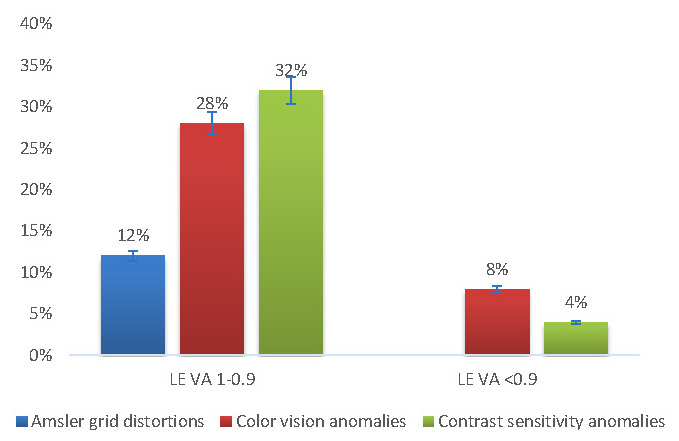
Left eye vision anomalies in relation to the visual acuity

80% of the patients had normal/ near-normal visual acuity (1 or 0.9 by convention) for the RE and 88% for the LE. PD patients with normal/ near-normal visual acuities displayed visual anomalies in both eyes, with highest incidences among the color vision anomalies (24% in the RE and 28% in the LE) and the contrast sensitivity anomalies (24% in the RE and 32% in the LE) (**Fig. 7**,**8**). 

When we applied the ttest to compare the visual anomalies in the same eye for the two different visual acuity classes (by convention), we observed statistically significant differences for the LE (*p* = 0.049). The same test revealed no significance for the RE (*p* = 0.069).

## Discussions

According to Cerri et al. (2019), even though men have a higher risk for developing PD, women display a more aggressive phenotype with higher mortality rates and accelerated progression patterns. Female patients who suffer from PD with dementia have lower survival rates than males. Moreover, female PD patients have later onset of motor symptoms with tremor predominance and postural instability [**5**]. Our study revealed that the gender distribution was similar with a normalized ratio of 62.5% in women and 58.82% in men, for the tremor-predominant type. Also, similar ratios were seen in the akinetic-rigid variant between women (37.5%) and men (35.29%). In our study, we compared both genders in relation to the postural instability and found that fewer women (-20%) presented postural instability than men.

Titova and coauthors (2017) commented on the diversity of PD subtypes, bringing out that the tremor predominant subtype could present fewer non-motor features than other subtypes. Also, these subtypes give heterogeneity to PD’s clinical picture and could originate from the differences of the cerebral neurotransmitters’ dysfunctions [**6**]. We found that the tremor predominant subtype displayed more frequent Amsler grid distortions and color vision anomalies than the other subtypes, while the contrast anomalies were more specific for the akinetic-rigid type.

According to some authors, postural instability is very common in PD and it becomes more frequent with disease progression, although it can be present at the time of diagnosis. The most severe complication of postural instability is the risk of falling and subsequent loss of independent life [**7**,**8**]. We showed that 36% presented postural instability, while only 64% had no instability. Fewer women (-20%) presented postural instability than men. The female gender showed fewer individuals with instability (8%) than those without instability (24%).

Opara et al. pointed out that the severity of the motor features and the cognitive decline are predictors for a faster progression of PD and subsequent disability [**9**]. 

According to Weil and colleagues, in a questionnaire addressed to PD patients, 78% reported at least one visual symptom. Another observation was that many PD patients had normal visual acuity, but impaired contrast sensitivity. Decreased contrast sensitivity is observed in central and peripheral locations. Regarding color vision anomalies, these correlated especially with axial motor symptoms [**10**]. Our study showed that PD patients with postural instability had more prevalent color vision anomalies (+14.29% with *p* = 0.027) and slightly more contrast disturbances (+4,76% with *p* = 0.068) than those without postural instability. Because postural instability is the result of axial musculature dysfunction, our results confirmed the observations reported by Weil et al. Moreover, postural instability alone contributes to the risk of falling and disability, but combined with sensory deficits like visual dysfunctions, these could further increase these risks. Moreover, we also noted that individuals with normal/ near-normal visual acuities presented visual anomalies, and especially color vision (24% in the RE and 28% in the LE) and contrast sensitivity anomalies (24% in the RE and 32% in the LE). We also observed statistically significant differences between the visual anomalies in the same eye for the two different visual acuity classes for the LE (*p* = 0.049).

It was reported that different genetic mutations linked to the pathophysiology of PD lead to specific sensory dysfunctions regarding color vision, visual hallucinations and cognitive status decline or even REM-sleep behavior disorder [**10**]. 

It has been suggested that the color vision anomalies could be specific to PD and therefore could be considered a diagnostic sign. Moreover, the cognitive decline could impact color discrimination in PD, if associated. In a pilot study, it was observed that light in the green spectrum could improve gait and reduce freezing phenomena. These observations could further increase our knowledge on the complexity of visual disturbances in PD [**11**,**12**]. Our study included only non-advanced stages of PD and we observed that the 4% classified in the 24-23 p. MMSE group (mild cognitive decline) displayed only contrast sensitivity anomalies. We also observed that the 30-29 p. MMSE group displayed the most frequent visual dysfunctions. This observation could be also because most of the subjects (48%) were classified in this MMSE class. However, since the cognitive decline appears in later stages of the disease evolution, our observation could confirm that the visual dysfunctions, and more specific the Amsler grid, Color vision and Contrast sensitivity anomalies, can appear in the early stages of PD.

Other authors suggested that the contrast sensitivity function is affected more as the disease progresses and that L-Dopa treatment could influence it [**13**,**14**].

It has also been suggested that the visual impairment in PD is linked to the general disability and gait disturbances of these patients [**15**].

Other noteworthy observations were that the color vision anomalies correlated with: right predominant parkinsonism, mean disease duration of PD and higher motor severity scores (HY 3 and UPDRS > 30 p.). On the other hand, contrast sensitivity anomalies correlated with: left predominant parkinsonism and lower motor severity scores (HY 1, HY 2 and UPDRS < 30 p.). Also, the Amsler grid distortions correlated with the motor evaluation scales of PD (HY and UPDRS). Another important aspect is that the mean PD duration correlated with anomalies in the ophthalmoscopic evaluation of both eyes. 

## Conclusions

In this second report we brought to light new data from this prospective study and showed important aspects of the visual dysfunctions and their correlation to PD. Because it is well established that PD is not just a motor disorder, but instead a complex condition that affects even the vision, we believe that the main concern of the clinical practitioner is to approach such patients in a multidisciplinary team in order to screen these hidden symptoms. Bridging the gap between healthcare specialists, and working together as a team, is vital for an early recognition of signs and prompt intervention in order to increase the quality of life.

Even though the study sample was relatively small, our observations could be important links in finding specific correlations between the motor features and the visual dysfunctions in PD, which could help us find new markers in recognizing this condition in its prodromal stage.

We believe that the multidisciplinary approach, personalized Medicine, and patient-tailored interventions are key aspects for *„Medicine 2.0”* in the future.

**Conflict of Interest Statement**

The authors state no conflict of interest. 

**Informed Consent and Human and Animal Rights statement**

Informed consent has been obtained from all the patients included in the study.

**Authorization for the use of human subjects**

Ethical approval: The research related to human use complies with all the relevant national regulations, institutional policies, it is in accordance with the tenets of the Helsinki Declaration and has been approved by the review board of “Iuliu Haţieganu” University of Medicine and Pharmacy, Cluj-Napoca, Romania. 

**Acknowledgements**

All authors contributed substantially for this manuscript. We thank all our patients who participated in this study and devoted their time for knowledge.

**Sources of Funding**

No source of funding to declare.

**Disclosures**

None.
